# Optical Studies and Transmission Electron Microscopy of HgCdTe Quantum Well Heterostructures for Very Long Wavelength Lasers

**DOI:** 10.3390/nano11071855

**Published:** 2021-07-19

**Authors:** Vladimir V. Rumyantsev, Anna A. Razova, Leonid S. Bovkun, Dmitriy A. Tatarskiy, Vladimir Y. Mikhailovskii, Maksim S. Zholudev, Anton V. Ikonnikov, Tatyana A. Uaman Svetikova, Kirill V. Maremyanin, Vladimir V. Utochkin, Mikhail A. Fadeev, Vladimir G. Remesnik, Vladimir Y. Aleshkin, Nikolay N. Mikhailov, Sergey A. Dvoretsky, Marek Potemski, Milan Orlita, Vladimir I. Gavrilenko, Sergey V. Morozov

**Affiliations:** 1 Institute for Physics of Microstructures of RAS, 603950 Nizhny Novgorod, Russia; annara@ipmras.ru (A.A.R.); tatarsky@ipmras.ru (D.A.T.); zholudev@ipmras.ru (M.S.Z.); kirillm@ipm.sci-nnov.ru (K.V.M.); utvlvas@ipmras.ru (V.V.U.); fadeev@ipmras.ru (M.A.F.); aleshkin@ipmras.ru (V.Y.A.); gavr@ipmras.ru (V.I.G.); more@ipmras.ru (S.V.M.); 2 Faculty of Radiophysics, Lobachevsky State University, 603950 Nizhny Novgorod, Russia; 3 LNCMI-EMFL, CNRS UPR3228, University Grenoble Alpes, University Toulouse, University Toulouse 3, INSA-T, 38042 Grenoble, France; evilra66it@gmail.com (L.S.B.); marek.potemski@lncmi.cnrs.fr (M.P.); milan.orlita@lncmi.cnrs.fr (M.O.); 4 Faculty of Physics, Lobachevsky State University, 603950 Nizhny Novgorod, Russia; 5 Resource Center for Nanotechnology, Saint-Petersburg University, 199034 Saint-Petersburg, Russia; zihertge@gmail.com; 6 Faculty of Physics, Lomonosov Moscow State University, 119991 Moscow, Russia; antikon@physics.msu.ru (A.V.I.); aurelia8002@gmail.com (T.A.U.S.); 7 Rzhanov Institute of Semiconductor Physics, Siberian Branch, Russian Academy of Sciences, 630090 Novosibirsk, Russia; remesnik@isp.nsc.ru (V.G.R.); mikhailov@isp.nsc.ru (N.N.M.); dvor@isp.nsc.ru (S.A.D.); 8 Advanced School of General and Applied Physics, Lobachevsky State University, 603950 Nizhny Novgorod, Russia; 9 Faculty of Mathematics and Physics, Institute of Physics, Charles University, KeKarlovu 5, 121 16 Prague 2, Czech Republic

**Keywords:** HgCdTe, stimulated emission, heterostructures

## Abstract

HgTe/CdHgTe quantum well (QW) heterostructures have attracted a lot of interest recently due to insights they provided towards the physics of topological insulators and massless Dirac fermions. Our work focuses on HgCdTe QWs with the energy spectrum close to the graphene-like relativistic dispersion that is supposed to suppress the non-radiative Auger recombination. We combine various methods such as photoconductivity, photoluminescence and magneto-optical measurements as well as transmission electron microscopy to retrofit growth parameters in multi-QW waveguide structures, designed for long wavelengths lasing in the range of 10–22 μm. The results reveal that the attainable operating temperatures and wavelengths are strongly dependent on Cd content in the QW, since it alters the dominating recombination mechanism of the carriers.

## 1. Introduction

At present, HgCdTe ternary alloys and HgCdTe heterostructures with quantum wells (QWs) have attracted a lot of interest in regard to their unique fundamental properties. Being a leading material for photoelectric detectors of the mid-infrared (IR) range [1], the growth technology for HgCdTe epitaxial structures has developed fairly well. HgTe itself has an inverted band structure; therefore, an arbitrary bandgap from 0 to 1.6 eV (latter value correspond to pure CdTe) can be obtained in ternary alloy [2]. HgCdTe QWs also allow the energy of interband transitions to be controlled in the range from a few to several hundreds of meV [3]. However, different combinations of x, y and QW thickness d can correspond to the same bandgap in Hg_1−x_Cd_x_Te/Cd_y_Hg_1−y_Te QW, while the energy spectrum of electrons and holes differs significantly for different parameters of the heterostructures. Knowing only the values of the bandgap at several temperatures is far from being sufficient to extract the QW parameters. It appears that slight features of the band diagrams can considerably affect the fundamental properties of HgCdTe QW heterostructures such as the dominating recombination mechanism of excess charge carriers. In turn, carrier lifetimes are of great importance in the narrow gap materials that are exploited for optoelectronic applications.

A lot of research is focused on HgTe/CdHgTe QWs with thicknesses close to the critical one of 6.3 nm (see for example Refs. [4,5,6,7]), at which the bandgap in the QW vanishes and the graphene-like energy-momentum law for carriers is realized. In particular, long-wavelength-stimulated emission (SE) was obtained recently in HgCdTe QW structures [8], paving the way to the development of interband lasers in the range of 20–60 μm. At the moment, this range is practically unavailable for quantum cascade lasers (QCLs) due to the strong lattice absorption in GaAs and InP materials [9], which are used for the vast majority of QCLs. In HgCdTe, the phonon frequencies are low enough to enter the 20–60 μm spectral region [10]. Recent research [11] suggests that the cornerstone of increasing the operating wavelength is to exploit narrow HgCdTe with low Cd content instead of wide HgCdTe QW and bulk-like layers used in previous works [12,13]. The advantage of the thin QWs is the Dirac-like carrier energy spectrum that suppresses the Auger recombination. However, the SE obtained from HgCdTe QW structures reveals a temperature limit, above which SE cannot be observed, and the limiting factors are yet to be firmly established. After increasing the QW width above the critical one, the HgCdTe/CdHgTe heterostructure turns into the 2D topological insulator, providing the edge states protected from the back-scattering [14]. Recent successes in detecting these states in samples with micron size [15] suggest that new types of “topological” THz detectors are on the verge of becoming reality [16]. Finally, the high mobility of nearly massless carriers is also the strong card for terahertz (THz) plasmonics. Both detection and emission can benefit from plasmon enhancement in the HgCdTe heterostructure, in the same way as in graphene [17].

The modern molecular beam epitaxy delivers high-quality heterostructures with in situ ellipsometric control of the composition and thickness of the layers [18]. However, for layers that are only several nanometers thick, accurate determination of the Cd content from ellipsometric data can be complicated. A number of unique fundamental effects mentioned earlier were investigated in single-QW HgTe/CdHgTe structures. Nevertheless, multi-QW structures are of prime interest for devices. At least 5–10 QWs are required for lasers and even more for detectors to achieve high quantum efficiency. Therefore, a high growth rate is needed, entailing the questions about uniformity and reproducibility of structures. Commonly used characterization techniques are often challenging for HgCdTe. In particular, there are not many papers on transmission electron microscopy of HgCdTe heterostructures [19,20], and the photoluminescence (PL) studies of HgCdTe in the very long wavelength range are also quite scarce [21,22]. Complications in PL studies are related to low efficiency of light emission and less sensitive detectors in the long wavelength region. In conjunction with a huge thermal background, it forces one to implement sophisticated modulated techniques [23] to detect emission and limits the time resolution/averaging rates of the experiment on account of typically slow thermal detectors.

This work presents a comprehensive complex characterization of the HgCdTe QWs via photoconductivity (PC), photoluminescence and magneto-optical measurements in mid- and far-infrared ranges as well as transmission electron microscopy (TEM) investigation of the structures under study. Revised data on QW parameters allowed us to calculate accurate band diagrams and carrier lifetimes that are in good agreement with the experiment, revealing the dominating recombination mechanism in structures under study. Analyzing the factors limiting the gain in the long-wavelength region, we improve the SE figures of merit. We demonstrate SE at wavelengths 22–16 μm persisting up to 80 K. The results suggest that SE is feasible at wavelengths more than 22 μm if the Auger recombination is suppressed.

## 2. Materials and Methods

All structures under study were grown by molecular beam epitaxy on semi-insulating GaAs (013) substrates with ZnTe (50 nm thick) and CdTe (10 μm thick) buffers [18]. To achieve light confinement, required for studying SE, an array of QWs was grown inside a widegap CdHgTe layer acting as the core of the dielectric waveguide. The QWs were placed inside the antinode of the TE_0_ mode of the waveguide to maximize the confinement factor. The discussion of the waveguide design and the examples of TE mode calculation can be found in Ref. [24]. The parameters of structures are presented in Table 1.

The study of the QW heterostructures performed in this work can be divided into the following steps:

- Experimental investigation of the optical properties of structures by different spectroscopy techniques: PC, PL and magneto-optical transmission measurements in mid- and far-infrared ranges. TEM was also used as a supplementary technique for some structures.
- Identification of the interband transitions on the spectra.
- Determination of the QW parameters (thickness and Cd content).
- Calculation of the band diagrams of structures, the rates of Auger and radiative recombination.
- Studies of SE and carrier density dynamics.
- Determination of the dominating recombination mechanism and the maximum temperature at which SE can be obtained.

The PC spectra were studied via Fourier transform infrared (FTIR) spectroscopy at liquid helium (4.2 K) and liquid nitrogen (77 K) temperatures. The samples were mounted at the end of the waveguide insert immersed into the Dewar vessel. The Vertex 80v FTIR spectrometer (BrukerOptik GmbH, Ettlingen, Germany)was used with globar as a radiation source with a potassium bromide beam splitter. KRS5 was used as a filter, providing a clear transmission window with no dips or other spectral features in the relevant spectral range. More information on the experimental setup can be found in Ref. [25]

The PL and SE spectra were obtained using the same FTIR spectrometer operating in the step scan mode. The sample was mounted inside a closed-cycle cryostat optically connected to the spectrometer. Mainly, a CO_2_ laser (with operating wavelength of 10.6 μm and pulse duration of ~100 ns) was used for pumping [26]. The parametric oscillator “Solar OPO” was exploited for several structures, when 2–2.3 μm pumping wavelength was appropriate. Details of such experiment can be found in Refs. [8,24]

Magneto-optical experiments were performed in the Faraday configuration in magnetic fields up to 11 T delivered by a superconducting coil. Samples were kept in the low-pressure helium exchange gas at the temperature of 4.2 K. Globar was used as a broadband source of infrared radiation. The radiation, analyzed by a Fourier transform spectrometer, was guided through the ZnSe entrance window of the sealed probe, delivered via light-pipe optics to the sample and detected by a composite silicon bolometer placed below the sample. The presented spectra are relative magneto-transmission, T_B_/T_0_, corrected for the field-induced changes in response of the bolometer. Details of this experiment can be found in Ref. [27].

To study the cyclotron resonance (CR) spectra in the far-infrared range, we used the simpler express method. The structure was mounted at the end of the waveguide insert inside a compact superconducting magnet. The magnetic field was varied from 0 to 3 T, while the radiation frequency was fixed at 526 GHz.We used the second harmonic of the sub-THz gyrotron operating at 263 GHz as the exciting radiation [28], providing highly stable intensity. A gapless HgCdTe epilayer was exploited as the detector, with sensitivity comparable to an InSb hot electron bolometer [29].

Carrier lifetimes were investigated via time-resolved PC measurements. The samples were excited with the parametric oscillator “Solar OPO” (SOLAR Laser Systems, Minsk, Belarus), providing 7 ns pulses either in the 2–2.3 μm or in the 7.7–9.5 μm range. A current preamplifier with 200 MHz bandwidth was used to recover the signal; therefore, the time resolution was limited only by the excitation pulse.

TEM lamella preparation was performed by FIB-SEM crossbeam station Zeiss Auriga Laser (Carl Zeiss Jena GmbH, Jena, Germany). The preparation followed the standard procedure for lift-out cross-section TEM lamella with optimized conditions [30]. The optimization of milling conditions had two goals: increasing the ion milling rate of the bulk sample and minimizing the ion beam damage to the thin area of the lamella. The preparation procedure included several steps. In the first step, the protective carbon-platinum bilayer was deposited onto the area of interest by electron-beam-induced chemical deposition with a 2kV@6nA electron beam. The thickness of the protective bilayer was about 600 nm. The second step was rough milling of two trapeziums on both sides of the area of interest, performed with a 30kV@4nA ion beam. This step resulted in the preparation of thick lamella with thickness of about 1 µm. Then, this lamella was removed from the sample and transferred to the TEM grid. The lamella thinning process was performed with a 30kV@20pA ion beam. The final polishing of the lamella surface was performed with 30kV@2pA. After polishing, the lamella was irradiated by low energy Ga^+^ ions with the 3kV@20pA beam to remove the residual damage of the surface layer. The thickness of the thin place of the lamella after the final polishing step was about 30 nm, and the area was about 1 µm^2^.Transmission electron microscopy was performed on aCarl Zeiss LIBRA200 MC microscope (Carl Zeiss Jena GmbH, Jena, Germany) operated at 200 kV.

To adjust the parameters of the grown structures, the experimental data were compared with the results of the calculations. The calculation method for band diagrams is based on the envelope function approximation [31].We used Burt–Foreman approximation [32] for a 8 × 8 Kane–Hamiltonian with parameters from Ref. [33]. The structures were grown on the crystallographic plane (013); therefore, the Hamiltonian has been modified accordingly [5,34]. The z-dependent components of the envelope functions were calculated by plane wave expansion. The model used to calculate the radiative recombination lifetimes is described in Ref. [35]. The threshold energies of Auger recombination were estimated numerically using the extremum search method as described in Ref [36]. Then, the Auger recombination rates were calculated with a microscopic model, taking into account the complex band dispersions and wavefunctions (calculated at the previous step), degenerate carrier statistics and screening effects. Details of the model can be found elsewhere [37].

## 3. Results

### 3.1. Transmission Electron Microscopy

**Figure 1:**
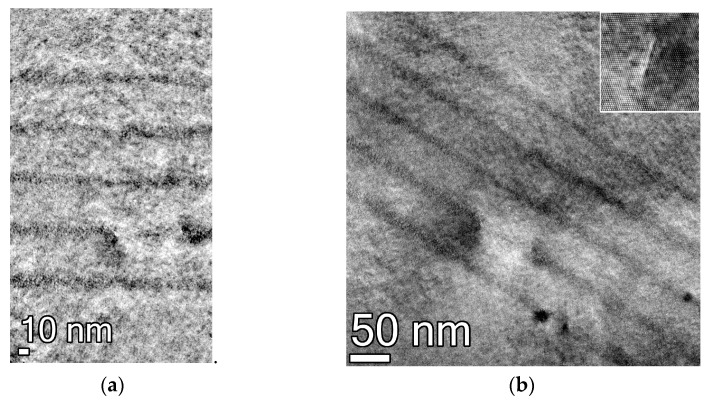
(**a**) Bright field micrograph of five Hg(Cd)Te QWs. The weak Z-contrast gives the widths of QWs d = 8–12 nm; (**b**) Overview of bright field micrograph with dark defected areas. A high-resolution image of a typical defected area is given in the inset.

Figure 1a presents a bright field micrograph, which shows a weak material contrast in the structure under study (#A0130). However, five QWs can be seen clearly, with the QW width being not less than 7.8 nm, the value provided by the in situ ellipsometry. High-resolution images of “dark areas” from the overview micrographs (Figure 1b) revealed defects similar to the ones studied in a recent work [38].Based upon the lateral average of contrast, we obtain the average width for QWs d = 8–12 nm. Notably, the QW width determined from TEM images in a recent work was also 2 nm larger than that extracted from the ellipsometry data [20]. One can anticipate that Cd is present in QW because pure HgTe QW would have an inverted band structure, resulting in a bandgap of 20 meV at the most, which is in poor agreement with the observed PC cutoff and SE wavelength (see below). However, the material contrast between QW layers and barriers makes it difficult to determine whether Cd is present in the QWs from TEM images.

Thus, the TEM studies reveal a tenable quality of structure and that QW width is not less than the targeted one, but does not facilitate determining the QW parameters with reasonable accuracy. Therefore, the optical studies were exploited for other structures.

### 3.2. Photoconductivity and Magnetooptical Studies

**Figure 2:**
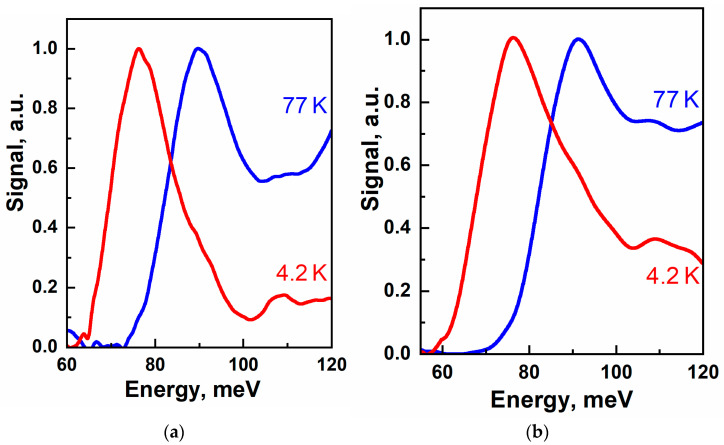
PC spectra of structures at 4.2 and 77K: (**a**) #A0130; (**b**) #B0225.

**Figure 3:**
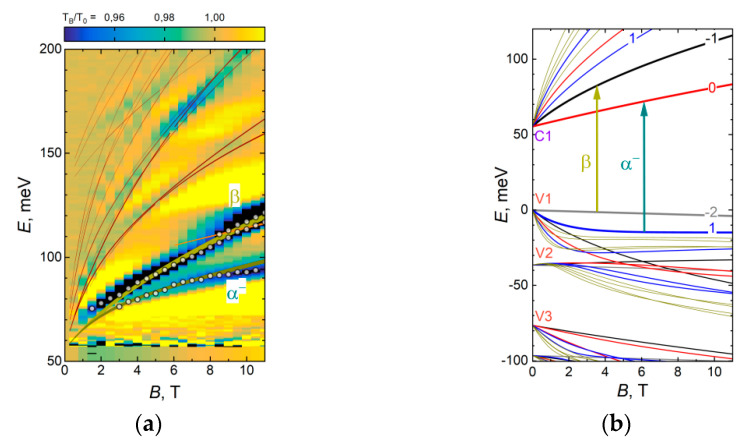
(**a**) Magneto-transmission of sample #A0130 at T = 4.2 K plotted in the form of false-color plots with circles indicating the peaks of the particular absorption lines. The solid lines in the overlay correspond to the theoretically expected energies of the transitions; specific transitions β and α− are labeled. The thickness of the line reflects the probability of transition. Theoretical calculations provide the best fit for the rectangular quantum well Hg_0.92_Cd_0.08_Te with a thickness of 7.8 nm.(**b**) Calculated Landau levels (LL) for a given structure; the arrows and Greek letters denote LL transitions, observed in the magneto-transmission spectra. Conduction and valence sub-bands are labeled on the left axis.

The PC spectra of structures demonstrate a fairly steep edge of the fundamental transition (see Figure 2). The width of the cutoff region (the difference between the photon energies corresponding to the 0.9 and 0.1 level of the PC signal maximum) is presented in Table 2 to have a figure of merit for the steepness of the edge. Note that the cutoff wavelength, corresponding to the fundamental interband transition from the first valence sub-band v1 to the first electron sub-band c1 (see Figure 3b), is shorter for 77 K for all structures. The cutoff wavelength shortening with temperature is a characteristic feature of HgTe/CdHgTe structures, with only the normal band ordering [39], and thus, immediately suggests that the QW parameters have considerable deviation from the ones provided by ellipsometry (see Table 1). It is well known that the HgTe/CdTe QWs wider than the critical thickness ~6.3 nm have inverted band structure, for which the bandgap shrinks [40] as the temperature is increased in contradiction to our experiment.

However, before correcting the structure parameters, one should exclude other factors that can alter the cutoff wavelength such as Burstein–Moss shift and/or the non-rectangular profile of the QW. Unfortunately, waveguide structures, i.e., structures with QWs buried inside a thick widegap layer (see Table 1), are challenging for transport measurements due to the poor quality of the contacts. Therefore, we opt for the cyclotron resonance effect to extract the carrier density in QWs. It also appeared that CR spectrum in conjunction with the interband magneto-absorption data provides an insight into structure parameters as well.

Figure 3a shows a false-color plot of relative magneto-transmission of structure#A0130 at 4.2 K.One may immediately recognize the most pronounced interband transitions α- and βin the energy range from 70 to 100meV, reported earlier for structures with single QWs [27,39]. Nonetheless, observation of additional absorption features at higher energies provides obvious benefits for comparison with calculated transitions, following the procedure described earlier [27].

It is not possible to adequately describe observed features for various combinations of QW profiles; thus, we have to assume that there is Cd in the QW. The best agreement with both PC and magneto-optical data was achieved for a QW width of 7.8 nm and 8% Cd in the QW. The accuracy of the extracted values can be estimated as 0.3 nm for QW width and 0.005 for Cd content. The lateral inhomogeneity values for QW parameters across the wafer reconstructed from ellipsometry during the growth are of the same order of magnitude.

Note that using the wider QW profile makes the agreement with the experiment worse. Therefore, it seems that the upper-bound estimate for the QW width of 12 nm, provided by the TEM image, stems from the quasi-exponential “tail” of Cd content distribution that appears at the top interface of the QW when Cd concentration is increased towards the barrier value y~0.65. This “tail” is clearly revealed in QW profile reconstructions, the example of which can be found in Ref. [41]. However, due to the “tail”, the profile of the QW is considerably altered only after Cd concentration goes as high as about 70% of the targeted Cd fraction in the barrier. Therefore, the low-energy states close to the QW bottom should not be affected with this “tail”, which is consistent with the experiment.

**Figure 4:**
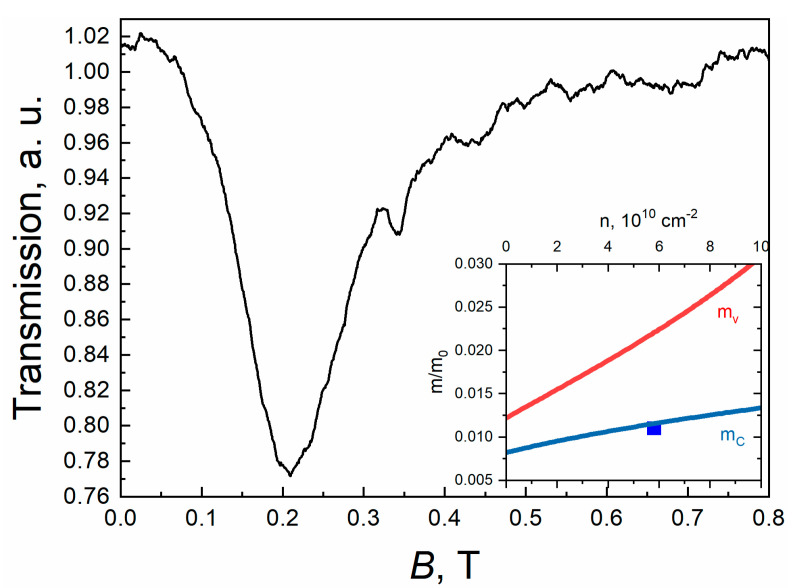
CR spectra and calculated cyclotron masses for electrons (m_e_) and holes (m_v_) vs. carrier density (m_0_ denotes the free electron mass) for sample #A0130 at T = 4.2 K. The measurement frequency is 526 GHz (corresponding to the photon energy of 2.17 meV).

One can also estimate the carrier concentration based on the magnetic field values that correspond to the onset of magneto-absorption lines (Figure 3a) as (5.3 ± 2) × 10^10^ cm^−2^. This estimate agrees well with the analysis of the area of the CR line (Figure 4), which gives the carrier density n = (5.8 ± 0.5) × 10^10^cm^−2^. The position of the CR line corresponds to the effective mass of 0.011m_0_ (m_0_ denotes the free electron mass), showing a good agreement with the calculation of cyclotron mass for electrons at n = 5.8 × 10^10^ cm^−2^.

Thus, magneto-optical studies (i) confirm the presence of Cd in the QW; (ii) show that a rectangular QW model adequately describes several low-energy sub-bands if the cadmium content is adjusted according to the cutoff wavelength observed in the PC spectrum; (iii) the QW thickness provided by ellipsometric data corresponds to the lower bound of the value that can be derived from TEM; however, this thickness agrees well with the optical transitions observed near the fundamental absorption edge.

## 4. Discussion

Using the discussed method of ex situ characterization, the parameters of QW—thickness and the concentration of Cd—were determined for a number of structures under study. To do this, the experimental data (the energies of the observed transitions at two temperatures) were compared with the results of the theoretical calculations performed in the framework of the Bert–Foreman model with the 8 × 8 Kane–Hamiltonian with temperature-dependent parameters. The values obtained are shown in Table 2. The general conclusion is that the QW thickness obtained by ex situ characterization agrees well with in situ ellipsometry, but Cd content differs considerably.

**Figure 5:**
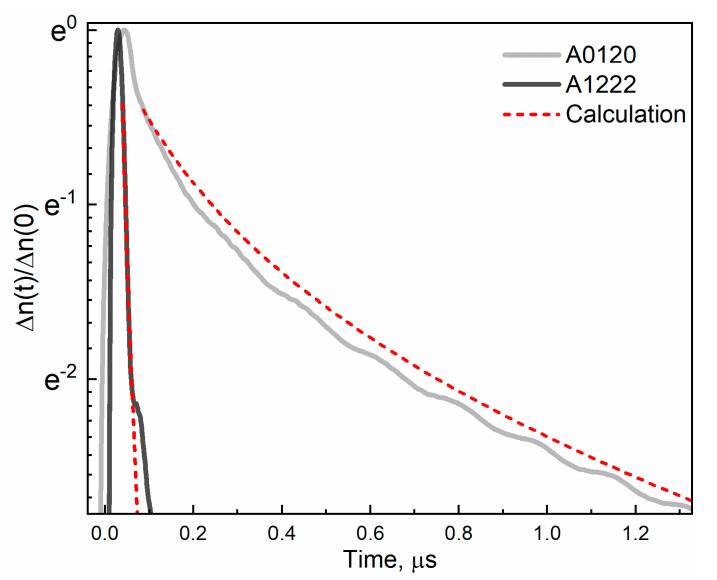
Carrier density kinetics (extracted from time resolved PC at 77 K) for str. #A0120 and #A1222 in comparison with the calculated relaxation kinetics. The initial non-equilibrium carrier density ∆n(0) is 9 × 10^10^ cm^−2^ for both structures.

The important consequence of different QW design is altering the threshold energy of Auger recombination even for the similar bandgap. The threshold energy of non-radiative Auger recombination (Table 2) with the participation of two electrons and hole (CCHC Auger process) was calculated based on the QW parameters [36] extracted from the ex situ measurements. As can be seen, the maximum “operating” temperature drops from 175 K in structure #A0120 to 100 K in structure #A1222, following a decrease in Auger threshold energy from ~40 meV to ~20 meV. This can be easily explained when analyzing the dynamics of the excess carrier density in these structures (Figure 5). The dramatic difference in carrier lifetimes is evident for these structures, despite the fact that PC cutoff wavelengths, i.e., the bandgaps, are very similar (Table 2). The experimental curves are accompanied by the theoretical ones, calculated with Auger and radiative recombination taken into account. As was shown earlier for structures analogous to str. #A0120 [35], in str. #A0120, the recombination is controlled by the radiative process. In contrast, according to the calculation for str. #A1222, the radiative recombination rate is at least an order of magnitude lower than for Auger recombination for the relevant carrier density range (10^10^–10^11^ cm^−2^). As a result, the experimental curve is well described by carrier relaxation driven only by the Auger process.

Note that the carrier dynamics are studied at liquid nitrogen temperature, which is considerably lower (kT ~ 7 meV) than the Auger threshold energy for structure #A1222. It can be concluded that even a tiny portion of carriers obtaining the kinetic energy above the threshold energy make a considerable impact on recombination. The possible reason for this is the additional heating of the distribution function introduced by the carriers remaining after the Auger process. Such electrons (or holes) carry a high kinetic energy (~E_g_) that is more likely to be distributed between the carriers than to be transferred to the lattice, leading to the heating of the entire electron–hole system.

In structure#A0130, the maximum “operating” temperature is less than one can expect from the empirical relation T_max_ ~ E_th_/2 observed for wider gap structures [42]. This fact can be attributed to the increasing role of free carrier absorption in the long wavelength range (the emission wavelength of #A0130 is two times longer than that of #A1222 or #A0120). To compensate for free carrier absorption, structure #B0225was grown with 10 QWs in the active region instead of 5 QWs for structures in the A series.

**Figure 6:**
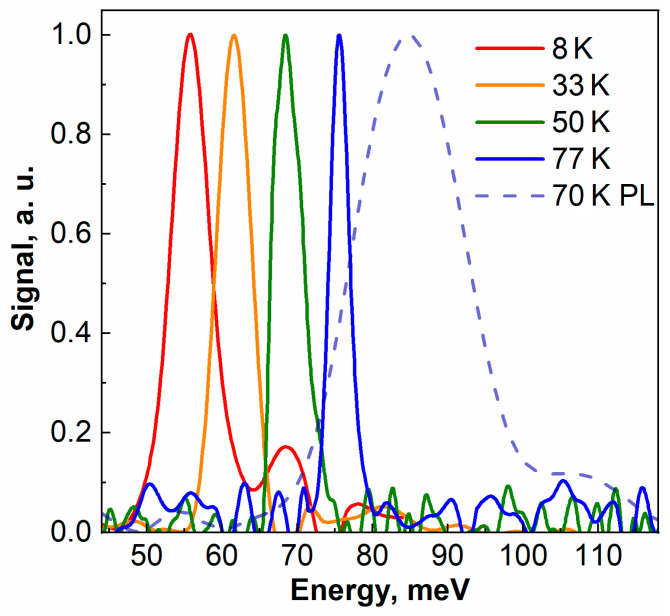
SE and PL spectra for str. #B0225. The SE spectra were obtained under pumping with pulsed CO_2_ laser (10.6 wavelength μm) at pumping intensity of 30 kW/cm^2^ (8 K and 33 K) and 100 kW/cm^2^ (50 K and 77 K). The PL spectrum was measured under cw excitation with 808 nm wavelength. All spectra are normalized to 1 for convenience.

As a result, SE at 22 μm was obtained from structure #B0225 with maximum “operating” temperature 80 K, thus showing improvement by 30 K over #A0130 in addition to a slight increase in the wavelength. Structure #B0225 demonstrates a threshold-like appearance of the emission line under pulsed excitation. Furthermore, the SE line FWHM is much less than that of the PL line observed under low-intensity cw pumping. The difference is especially visible at high temperatures, because the PL spectrum broadens in proportion to kT, while the FWHM of the SE line remains similar in a wide temperature range (see Figure 6). It should be noted that after the onset (which takes place at the threshold pumping intensity listed in Table 2), the FWHM of the SE line tends to increase with the pumping intensity, which can be attributed to the broadening of the gain spectrum and carrier heating [8]. In Figure 6, the SE spectra at 77 K are the narrowest because they correspond to the pumping intensity slightly above the threshold, while at 8 K, the spectrum was measured at a pumping intensity of 30 kW/cm^2^, which is more than seven times higher than the threshold value (4 kW/cm^2^, see Table 2). For the same reason, the SE spectrum at 30 K is narrower than that at 8K, and the SE spectrum at 50 K is wider than that at 77 K. Typical FWHM of the SE spectrum slightly above the threshold is 2–4 meV for all structures and temperatures [8,26,42].

From comparing the refined parameters of the studied structures (Table 2), it can be concluded that in order to further increase the temperature and the wavelength at which SE can be obtained in HgCdTe QW heterostructures, it is necessary to grow structures with narrower QWs containing lower concentrations of Cd, since they provide higher threshold energy for the Auger process. It should be noted that both narrowing of QWs and increases in their number can degrade the steepness of the absorption edge. Within A series, the highest blurring of the PC cutoff is observed for 3.2-nm-thick QWs in str. #A0120. Structures with wider QW (6–8 nm) have steeper long-wavelength edges. In structure #B0225, with comparable thickness of 7 nm, the steepness is worse, approaching that of str. #A0120. This can be attributed to a two-fold increase in the number of QWs.

## 5. Conclusions

It can be concluded that a slight variation in Hg_1−x_Cd_x_Te/Cd_y_Hg_1−y_Te QW parameters can have a strong impact on carrier recombination, even if the bandgap is not considerably altered. Consequently, one has to precisely control the QW width and Cd content when manufacturing structures for light sources or detectors in the long wavelength range. A multi-method approach presented in this work showed that a single characterization technique can lack the data to accurately extract the parameters of the grown structure. Photoconductivity spectroscopy combined with magneto-transmission or TEM becomes a powerful tool to analyze the QW design. While TEM reveals the actual width of the QW, and magneto-optics readily indicates the non-zero Cd content in it, the best accuracy is achieved from PC analysis once the initial approximation for structure parameters is worked out from the other techniques. The complications of PC spectroscopy are related to noisy signal in the case of poor quality of contacts and the parasitic features in the spectra resulting from artifacts.

PL spectroscopy of the interband transitions is challenging and requires Auger recombination to be suppressed considerably. The first step to do so is to exploit narrower QW, increasing the Auger recombination threshold energy. The ultimate case is binary HgTe/CdHgTe QWs. However, it should be underlined that the direct measurement of carrier lifetimes is needed to rigorously associate the enhancement in SE with structure parameters. Recent calculations suggest that screening effects can alter the optimal QW design to minimize the Auger recombination rates [37] at high carrier densities, which is yet to be confirmed in the experiment.

This work shows that the quenching temperature of stimulated emission drops more rapidly with a decrease in the bandgap than one would expect from the Auger recombination threshold. This finding can be attributed to the increasing role of free carrier absorption that requires higher carrier density for achieving overall gain. Higher carrier density results in band filling up to the point where the threshold energy of the Auger process is reached by many carriers and the onset of rapid non-radiative recombination takes place. In addition, some carrier heating is introduced by pumping, which is also triggering the Auger process. It was shown earlier [8] that raising the pumping power can quench SE, even after the onset. Therefore, one needs to increase the Auger threshold energy considerably above the kinetic energy at the quasi-Fermi levels and keep the carrier density at the onset of the amplification moderate. Since higher carrier density means a less favorable ratio between Auger and radiative recombination rates, it is preferable to achieve higher gain by increasing the number of QW in the active region. As shown in this work, the uniformity of structures remains tenable when the number of QW is increased up to 10 and no dramatic blurring of the band edges occurs.

## Figures and Tables

**Table 1:** Targeted parameters of structures under study: the thickness of the waveguide layer (D), Cd content in waveguide and barriers (y), QW thickness (d) and Cd content in (x) by in situ measurements (x (and y) values are defined as in Hg_1−x_Cd_x_Te), number of QWs (N).

Sample No.	D (µm)	y	d (nm)	x	N
A0120	2	0.58	3.65	0	5
A0130	8	0.66	7.8	0	5
B0225	9	0.65	6.8	0	10

**Table 2:** Parameters of structures under study: QW thickness (d) and Cd content in QWs (x) from ex situ characterization, PC cutoff wavelength at 4.2 K and 77 K, SE wavelength at 8 K (λ_SE_) and threshold (I_th_), critical temperature of SE quenching (T_max_), threshold energy of CCHC Auger recombination (E_th_), the PC cutoff width at 4.2 K (W), residual carrier density at 4.2 K (* 77 K, determined via Hall effect measurements) (n_dark_).

Sample No.	d	x	PC Cutoff, μm		λ_SE_, μm	I_th_, kW/cm^2^	T_max_, K	E_th_, meV	W, meV	N_dark,_ 10^10^ cm^−2^
	Ex Situ (±0.3)	Ex Situ (±0.005)	4.2 K	77 K						
A0130	7.8	0.08	17.9	15.2	20.3	5	50	14.7	7.6 ± 1.6	5.8 ± 0.6
B0225	6.9	0.067	18.3	15	22	4	80	20.1	8.6 ± 0.8	6.2 ± 0.6
A1222	6.1	0.945	10.5	9	11	0.1	100	19	3.4 ± 0.5	6.0 ± 0.6
A0120	3.2	0	10.5	8.87	10.2	0.13	175	42.6	9.6 ± 1.4	0.15 ± 0.03 *

## Data Availability

The data presented in this study are available on request from the corresponding author.
